# AMP Kinase Activation is Selectively Disrupted in the Ventral Midbrain of Mice Deficient in Parkin or PINK1 Expression

**DOI:** 10.1007/s12017-018-8517-7

**Published:** 2018-11-08

**Authors:** Liting Hang, John Thundyil, Geraldine W. Y. Goh, Kah-Leong Lim

**Affiliations:** 1 National Neuroscience Institute, 11 Jalan Tan Tock Seng, Singapore, 308433 Singapore; 2 NUS Graduate School for Integrative Sciences and Engineering, National University of Singapore, 21 Lower Kent Ridge, Singapore, 119077 Singapore; 3 Department of Physiology, Yong Loo Lin School of Medicine, National University of Singapore, 2 Medical Drive, MD9, Singapore, 117593 Singapore; 4 Neuroscience & Behavioral Disorders Program, Duke-NUS Medical School, 8 College Road, Singapore, 169857 Singapore

**Keywords:** AMP kinase, Mitochondria, Parkinson’s disease, Parkin, PGC-1α, PINK1

## Abstract

Parkinson’s disease (PD) is a prevalent neurodegenerative movement disorder that is characterized pathologically by the progressive loss of dopaminergic (DA) neurons in the substantia nigra pars compacta (SNpc) of the midbrain. Despite intensive research, the etiology of PD remains poorly understood. Interestingly, recent studies have implicated neuronal energy dysregulation as one of the key perpetrators of the disease. Supporting this, we have recently demonstrated that pharmacological or genetic activation of AMP kinase (AMPK), a master regulator of cellular energy homeostasis, rescues the pathological phenotypes of *Drosophila* models of PD. However, little is known about the role of AMPK in the mammalian brain. As an initial attempt to clarify this, we examined the expression of AMPK in rodent brains and found that phospho-AMPK (pAMPK) is disproportionately distributed in the adult mouse brain, being high in the ventral midbrain where the SN resides and relatively lower in regions such as the cortex—reflecting perhaps the unique energy demands of midbrain DA neurons. Importantly, the physiologically higher level of midbrain pAMPK is significantly reduced in aged mice and also in Parkin-deficient mice; the loss of function of which in humans causes recessive Parkinsonism. Not surprisingly, the expression of PGC-1α, a downstream target of AMPK activity, and a key regulator of mitochondrial biogenesis, mirrors the expression pattern of pAMPK. Similar observations were made with PINK1-deficient mice. Finally, we showed that metformin administration restores the level of midbrain pAMPK and PGC-1α expression in Parkin-deficient mice. Taken together, our results suggest that the disruption of AMPK-PGC-1α axis in the brains of individuals with Parkin or PINK1 mutations may be a precipitating factor of PD, and that pharmacological AMPK activation may represent a neuroprotective strategy for the disease.

## Introduction

Parkinson’s disease (PD) is a prevalent neurodegenerative disease that affects millions of predominantly elderly individuals worldwide. Clinically, the disease is attended by a constellation of motoric deficits that includes bradykinesia (slowness in movements), rigidity, and tremor that arise as a result of striatal dopamine depletion due to the progressive loss of midbrain dopaminergic (DA) neurons in the substantia nigra pars compacta (SNpc). Although a subject of intense research, the molecular events underlying PD pathogenesis remain not well understood. Nonetheless, a broad range of studies conducted over the past few decades have implicated aberrant mitochondrial homeostasis as a key culprit (Lim and Zhang 2013). Further supporting this, several groups have found that disease-associated mutations in PD-linked genes, especially *Parkin* and *PINK1*, exert profound effects on mitochondrial quality control. A corollary to this is that bioenergetics failure may represent a key driver of neurodegeneration in PD (Mattson and Arumugam 2018). This proposition is intuitively logical as post-mitotic neurons particularly those with vast axonal field are known to require high energy to support their operations, which include the active transportation of components (including mitochondria) towards metabolically demanding synaptic terminals that are distally located. In particular, SNpc DA neurons are characterized by unusually large number of axon terminals and axonal arborization, which overall increase their demand for ATP (Pissadaki and Bolam 2013). Indeed, Pacelli and colleagues recently demonstrated that elevated mitochondrial bioenergetics and axonal arborization underlie the selective vulnerability of SNpc DA neurons to degeneration in PD (Pacelli et al. 2015). Thus, strategies that improve the bioenergetics status of DA neurons may potentially be beneficial.

AMP kinase (AMPK) is a key cellular nutrient and energy sensor that is activated in response to falling energy supply, e.g., ATP depletion or glucose starvation (Hardie et al. 2012). It exists as a heterotrimer comprised of a catalytic α subunit and two regulatory subunits: β and γ. AMPK becomes activated upon phosphorylation of the α subunit on threonine-172. The major upstream kinases identified to be responsible for AMPKα Thr172 phosphorylation are liver kinase B1 (LKB1) and calcium/calmodulin-dependent kinase kinase β (CaMKKβ), although the enzyme can also be activated by pharmacological means via administration of compounds like 5-aminoimidazole-4-carboxamide ribonucleoside (AICAR) and metformin (Zhou et al. 2001). When activated, AMPK switches on catabolic processes that favor energy conservation and restoration, while simultaneously switching off energy-consuming processes (Hardie et al. 2012). Given the critical role of AMPK in regulating intracellular energy metabolism as an adaptive response to energy depletion, it is perhaps not surprising to note that AMPK has profound influence on mitochondrial homeostasis amidst a plethora of metabolic events that it controls. It is well documented that AMPK works through peroxisome proliferator-activated receptor gamma coactivator 1-alpha (PGC-1α) to promote biogenesis of mitochondria (Zong et al. 2002). Given this, AMPK activation may represent a suitable strategy to improve the bioenergetic status of DA neurons. Supporting this, we have recently demonstrated that the activation of AMPK mitigates mitochondrial abnormalities and DA neuronal dysfunction in *Drosophila* genetic models of PD (Ng et al. 2012), and in a PGC-1α-dependent manner (Ng et al. 2017). However, whether this strategy works in mammalian models remain to be tested. Notably, little is known to date about the role and regional distribution of AMPK in the mammalian brain. To clarify this, we found here that phospho-AMPK (pAMPK) is regionally distributed in the adult mouse brain in a manner that appears to reflect the unique energy demands of midbrain DA neurons, the expression pattern of which is mirrored by its downstream target PGC-1α. Importantly, the physiologically higher level of midbrain pAMPK is significantly reduced in Parkin-deficient mice, which can be restored by metformin treatment. Similar observations were made with PINK1-deficient mice. Taken together, our results suggest that deficient expression of pAMPK in Parkin or PINK1 mutant midbrains may contribute to bioenergetics impairments in DA neurons and precipitate PD, and that pharmacological AMPK activation may represent a neuroprotective strategy for the disease.

## Materials and Methods

### Antibodies

The following antibodies from Cell Signaling (USA) were used: monoclonal α-pAMPK, α-tyrosine hydroxylase and polyclonal α-TFAM. Antibodies from other manufacturers include monoclonal α-AMPK and polyclonal α-PGC-1α (Abcam, UK), monoclonal α-PRK8 (Signet/Covance, USA), α-beta-actin (Sigma, USA), α-PARIS (Merck Millipore, USA), α-mouse horseradish peroxidase and α-rabbit horseradish peroxidase (GE Healthcare, UK).

### Preparation of Mouse Brain Lysates

All animal-related studies were approved by and conformed to the guidelines of the Institutional Animal Care and Use Committee of TTSH-NNI (TNI-15-01-005). Seven 4–6-month-old wild-type C57B6 male mice or Parkin null mice (Von Coelln et al. 2004) (kind gift from Dr. T. Dawson, Johns Hopkins Medicine), and four 7-month-old PINK1 null mice were sacrificed via cervical dislocation. Male mice only were used in our study as we wish to exclude the potential effects of estrus cycle on AMPK activation, which may confound our observations. A summary of the age and number of the various mice examined in this study is provided in Table 1. To prepare the lysates, the brain was dissected into different regions (cerebellum, cortex, dorsal midbrain, hippocampus, olfactory bulb, striatum, and ventral midbrain) and homogenized in lysis buffer (PBS with 1% Triton X-100, cold or 1% SDS, room temperature, phenylmethylsulfonyl fluoride, aprotinin, Roche, USA), phosphatase inhibitor cocktail 2 and 3 (Sigma, USA) with a plastic homogenizer. Each brain section was placed in a separate microfuge tube. Lysates were sonicated and centrifuged at 13,500 rpm at 4 °C (for 1% Triton X-100) or 25 °C (for 1% SDS) for 15 min before the supernatant was collected. Protein concentration was quantified using Bradford protein assay and equal amount of lysates was loaded for western blot analysis.

**Table 1:** Type, age, and sample size of the animals examined

Figure nos.	Type of mice	Age (months)	Sample size
1	Wild-type	4–6	9
2	Wild-type	4–6	9
3a	Wild-type	2	4
	Wild-type	20	4
3b and c	Wild-type	4–6	5
4a	Parkin null	4–6	7
4b	PINK1 null	7	4
5	Parkin null (control)	9	4
	Parkin null (0.1% metformin)	9	4

### Metformin Treatment of Animals

Four 5-month-old adult Parkin null male mice were fed with 0.1% w/w metformin feed (Harlan^®^ Labs 0.1% Metformin Diet) while another group of four age-matched adult Parkin null male mice were fed with control feed (Harlan^®^ Labs Purified Diet) for a period of four months (ad libitum) before they were sacrificed. The brains from these two groups of mice were harvested and dissected for immunoblot analysis according to the methods described above.

### Statistical Analysis

Statistical analyses were performed using the Student’s two-tailed unpaired *t*-test (*p < 0.05, **p < 0.001). All data were expressed as mean (SEM) generated from at least three independent experiments.

## Results

### The Ratio of Phosphorylated AMPK/AMPK is Upregulated in the Ventral Midbrain Region of Normal Mice

**Fig. 1:**
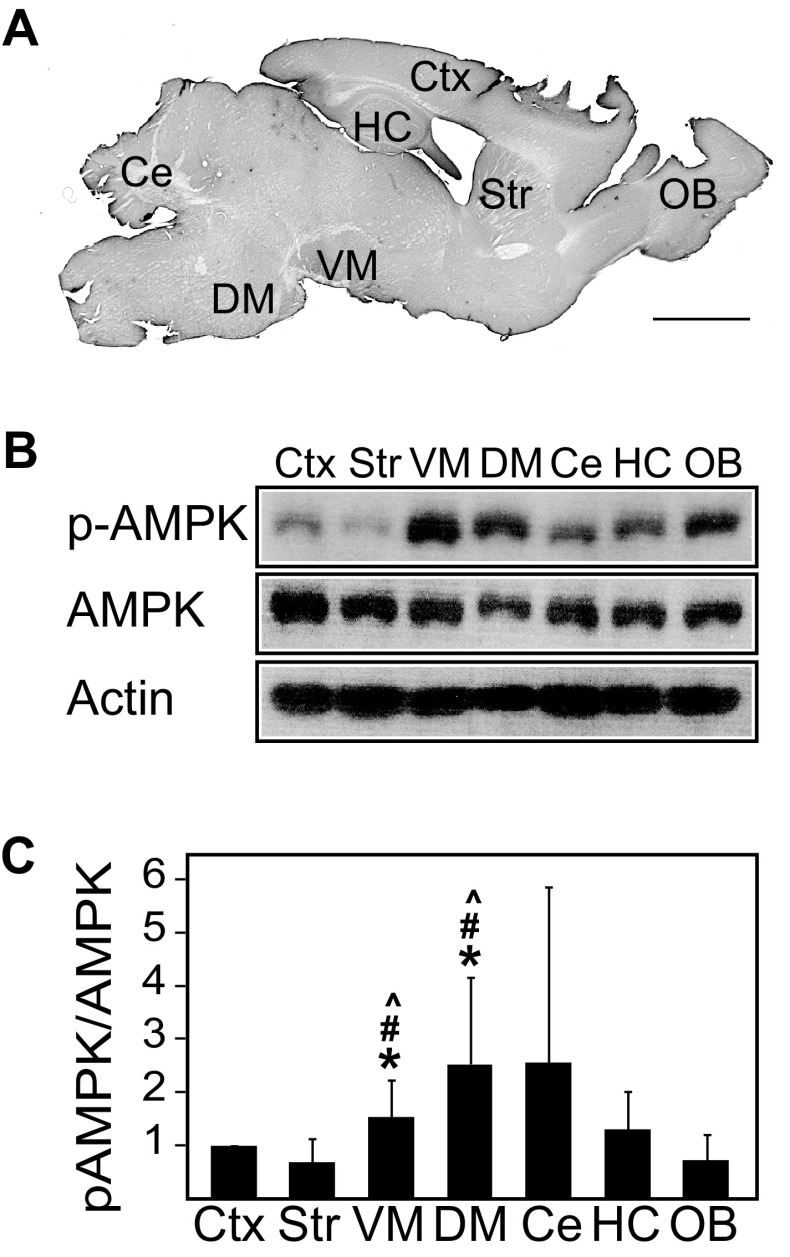
Phosphorylated AMPK/AMPK ratio varies across the different brain regions of normal mice. **a** Sagittal section of wild-type mouse brain depicting the location of the respective dissected brain regions (*Ctx* cortex, *Str* striatum, *VM* ventral midbrain, *DM* dorsal midbrain, *Ce* cerebellum, *Hc* hippocampus, *OB* olfactory bulb), scale bar = 2 mm. **b** Immunoblots showing the levels of pAMPK and AMPK across the different brain regions prepared from 4- to 6-month-old wild-type mice. The blots above were stripped and reprobed with anti-actin antibody to reflect loading variations. **c** Bar graph showing the average densitometric value of pAMPK/AMPK ratio in these regions (*n* = 9). (*p < 0.05 compared to Ctx, ^#^*p* < 0.05 compared to Str, ^*p* < 0.05 compared to OB)

**Fig. 2:**
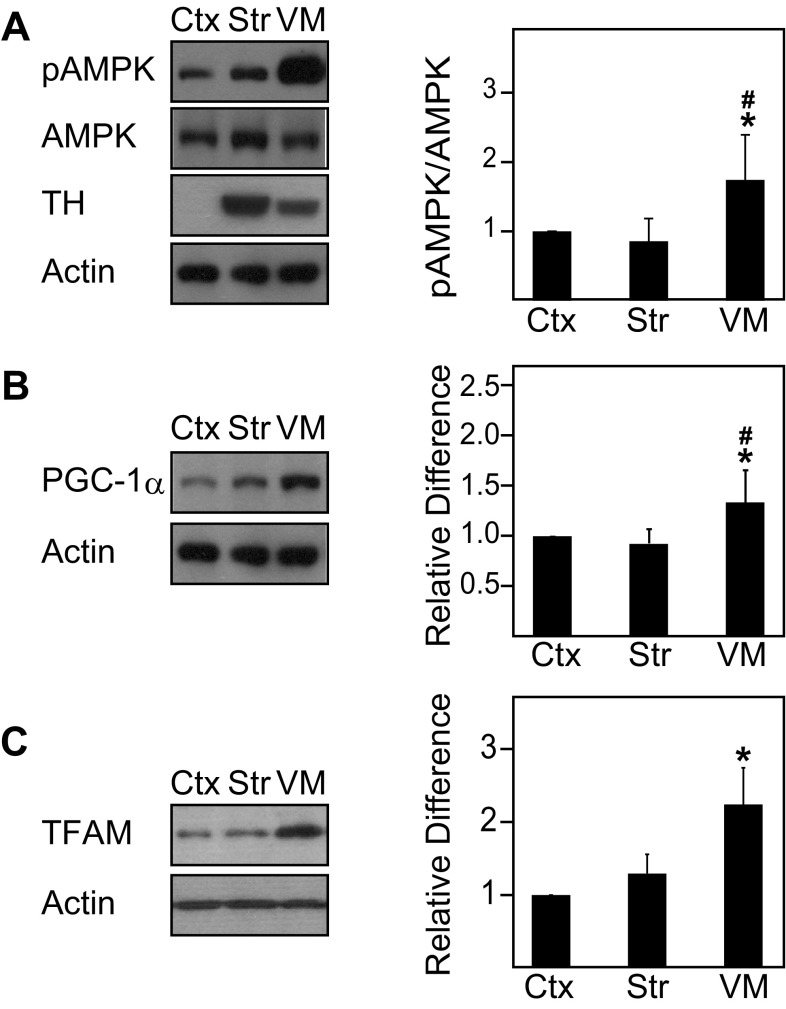
Phosphorylated AMPK/AMPK ratio, PGC-1α, and TFAM expression are significantly upregulated in the ventral midbrain region. **a** Left, immunoblots showing the levels of pAMPK, AMPK, and tyrosine hydroxylase (TH) in three selected regions (i.e., Ctx, Str, and VM) of the wild-type mouse brain. Right, bar graph showing the average densitometric value of pAMPK/AMPK ratio in these regions (*n* = 9). **b** Same as **a** except that PGC-1α expression is shown here (*n* = 5). **c** Same as **a** except that TFAM expression is shown here (*n* = 5). The blots above were stripped and reprobed with anti-actin antibody to reflect loading variations. (*p < 0.05 compared to Ctx, ^#^*p* < 0.05 compared to Str)

**Fig. 3:**
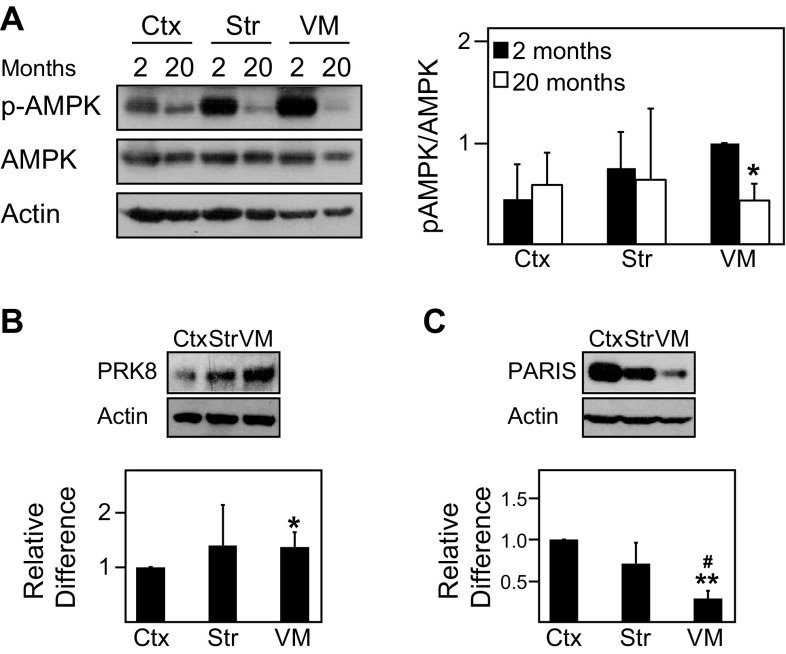
Phosphorylated AMPK/AMPK ratio is significantly reduced in the ventral midbrain region of aged wild-type mice. **a** Left, immunoblots showing the levels of pAMPK and AMPK in the same selected brain regions of aged wild-type mice (20 month old,* n* = 4) compared to young wild-type mice (2 month old, *n* = 4). Right, bar graph showing the average densitometric value of pAMPK/AMPK ratio in these regions. **b** Top, immunoblots showing the levels of Parkin in same selected brain regions of wild-type mice. Bottom, bar graph showing the average densitometric value of Parkin levels in these regions (*n* = 5). **c** Same as **b** except that PARIS expression is shown here (*n* = 5). The blots above were stripped and reprobed with anti-actin antibody to reflect loading variations. (*p < 0.05, **p < 0.001 compared to Ctx, ^#^*p* < 0.05 compared to Str)

To date, the expression and activity of AMPK in the normal brain remains poorly characterized. To address this, we examined the levels of phosphorylated AMPK (pAMPK) and total AMPK in different regions of the adult mouse brain. Interestingly, we found that the ratio between pAMPK and AMPK differs across various regions of the mouse brain, being significantly higher in the ventral midbrain region relative to the cortex and other regions (Figs. 1, 2a). Given that the ventral midbrain contains nigral DA neurons, it is tempting to think that its high pAMPK/AMPK ratio may reflect the well-known high energy demands of this group of neurons. However, the phenomenon may be confined to the cell body of DA neurons as the pAMPK/AMPK ratio is comparatively reduced in striatal regions where the terminals of these neurons reside (Figs. 1, 2a). Consistent with this profile, the level of PGC-1α, a downstream target of AMPK, correlates well with the level of AMPK activity in the different brain regions (Fig. 2b). As AMPK is also known to promote the expression of nuclear-encoded mitochondrial proteins through the activation of transcription factors, we measured the expression of TFAM and found that the level of this important nuclear-encoded mitochondrial transcription factor is similarly elevated in the ventral midbrain region relative to other regions examined (Fig. 2c). As age is an unequivocal risk factor for the development of PD, we also examined whether the level of AMPK activity in the brain may be affected by the aging process. Interestingly, although we found that the pAMPK/AMPK ratio exhibits a general trend of reduction with age, the decrease is selectively significant in the ventral midbrain of 20-month-old mice relative to their younger 2-month-old counterparts (Fig. 3a).

### The Expression of Phosphorylated AMPK is Significantly Reduced in the Ventral Midbrain of Aged, Parkin-Deficient, and PINK1-Deficient Mice

**Fig. 4:**
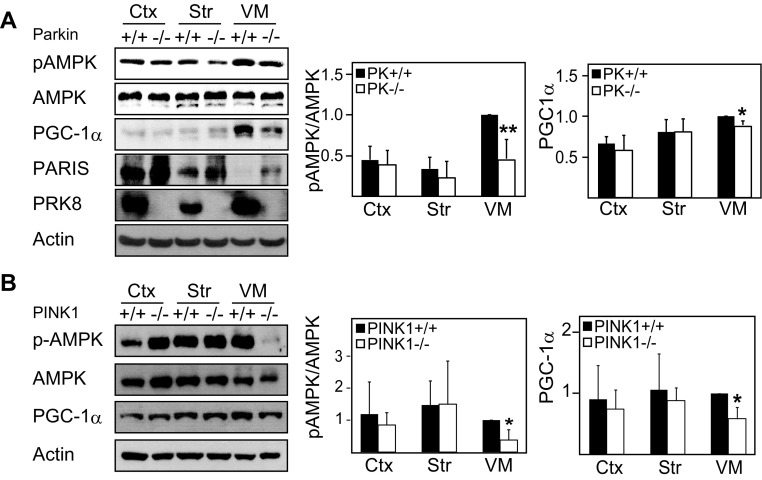
Phosphorylated AMPK/AMPK ratio is significantly reduced in the ventral midbrain region of Parkin null or PINK1 null mice brain. **a** Left, immunoblots showing the levels of pAMPK, AMPK, PGC-1α, PARIS, and Parkin in the three selected brain regions of 4–6-month-old wild-type (+/+) and Parkin null (−/−) mice. Right, bar graphs showing the average densitometric value of pAMPK/AMPK ratio and PGC-1α level in these regions (*n* = 7). (B) Left, immunoblots showing the levels of pAMPK, AMPK, and PGC-1α in the three selected brain regions of 7-month-old wild-type (+/+) and PINK1 null (−/−) mice. Right, bar graphs showing the average densitometric value of pAMPK/AMPK ratio and PGC-1α level in these regions (*n* = 4). The blots above were stripped and reprobed with anti-actin antibody to reflect loading variations. All the experiments above were repeated at least three times. (*p < 0.05 and **p < 0.001 compared to respective wild-type region)

Our previous report demonstrating that AMPK activation mitigates the pathological phenotypes of *Drosophila* Parkin null mutants (Ng et al. 2012) suggests that AMPK and Parkin may be functionally connected. To further investigate the potential relationship between AMPK and Parkin, we examined the expression of Parkin in cortical, striatal, and ventral midbrain regions harvested from normal mouse brain and found that Parkin expression correlates with the levels of pAMPK (Fig. 3b), which is interesting. Correspondingly, the expression of PARIS, a negative regulator of PGC-1α transcription, in the ventral midbrain is reverse that of pAMPK and Parkin (Fig. 3c), which may also explain the observed enhanced PGC-1α level in this region (Fig. 2b). Importantly, when we repeated the analysis with brain regions derived from Parkin null mice (4–6 months old), we recorded a significant and selective decrease in the pAMPK/AMPK ratio in the ventral midbrain region of these mutant mice (Fig. 4a). Alongside this, we also observed an associated significant reduction in the level of PGC-1α in the ventral midbrain region of Parkin-deficient mice relative to their control counterparts (Fig. 4a). Consistent with the report by Shin et al. (2011), we found that PARIS expression is upregulated in the ventral midbrain of these mutant mice (Fig. 4a). As Parkin and PINK1 functions are intimately interwoven, we also examined the above phenomena in PINK1-deficient mice. We found that PINK1-deficient mice, like their Parkin counterparts, exhibit selective reduction in the pAMPK/AMPK ratio as well as PGC-1α expression in their ventral midbrain region (Fig. 4b). Taken together, our results indicate a selective impairment of the AMPK-PGC-1α axis in Parkin- and PINK1-deficient ventral midbrain neurons.

### The Reduced Ratio of Phosphorylated AMPK/AMPK in the Ventral Midbrain Region of Parkin-Deficient Mice is Rescued by Metformin Treatment

**Fig. 5:**
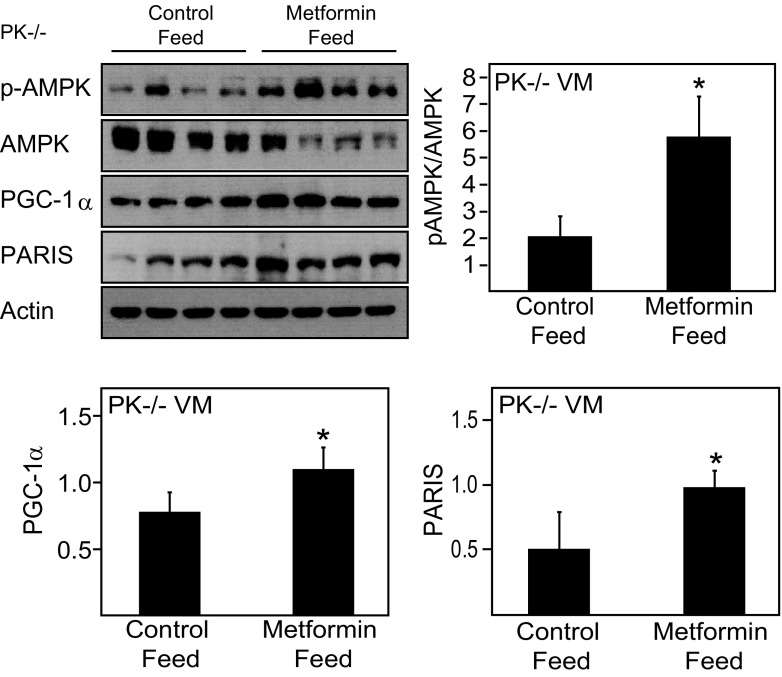
Metformin treatment selectively restores midbrain pAMPK/AMPK in Parkin null mice. Immunoblots show the levels of pAMPK, AMPK, PGC-1α, and PARIS in the ventral midbrain (VM) tissues of 9-month-old Parkin null mice in the absence or presence of metformin treatment (*n* = 4). Bar graphs show the average densitometric value of pAMPK/AMPK ratio, PGC-1α, and PARIS in untreated and treated Parkin null mice (*p < 0.05 compared to control diet)

Given the observed reduction in the levels of pAMPK and PGC-1α in the midbrain of Parkin-deficient mice, we wished to find out whether pharmacological activation of AMPK could restore their deficiency. We therefore administered Parkin null mice with metformin (an AMPK activator) via oral feed. Consistent with a report by Martin-Montalvo et al. (2013), metformin treatment for a period of 4 months increases AMPK activity in the liver of these mice, suggesting that metformin has been effectively metabolized, although no significant changes in body mass were recorded, other than a slight increase in mass in the metformin-treated group at 2-month post-treatment (not shown). Importantly, treatment of these mice with metformin results in a dramatic and significant rescue of the level of phosphorylated AMPK that is otherwise deficient in the ventral midbrain region of Parkin null mice (Fig. 5). Curiously, we documented an increased level of PARIS in the metformin-treated group relative to their untreated counterparts (Fig. 3). Notwithstanding this, we found that the level of PGC-1α is enhanced in the ventral midbrain region of metformin-treated Parkin null mice (Fig. 5), suggesting that pharmacological AMPK activation alone is sufficient to restore the deficient AMPK-PGC-1α pathway in Parkin null mice.

## Discussion

As an initial attempt to clarify the role of AMPK in the mammalian brain, we have conducted a regional expression profiling of pAMPK and AMPK in the brains of normal and Parkin/PINK1 null mice in the present study. In agreement with published literature documenting the high metabolic activities of SNpc DA neurons, we found that the pAMPK/AMPK ratio (which reflects the cellular energy requirements) is significantly higher in the ventral midbrain of the rodent brain compared to various other regions examined. Interestingly, this physiological upregulation of midbrain pAMPK/AMPK ratio is selectively downregulated in Parkin and PINK1 null mice. Not surprisingly, the expression of PGC-1α, a downstream target of AMPK, essentially follows the profile of phosphorylated AMPK. Although somewhat preliminary, it is tempting to suggest from our findings here that impaired AMPK/PGC-1α activation may contribute to DA neurodegeneration in Parkin- and PINK1-related PD cases, particularly in view of recent reports highlighting that SNpc DA neurons are characterized by unusually high energy demands (Pacelli et al. 2015; Pissadaki and Bolam 2013). However, we are cognizant that neither Parkin nor PINK1 null mice exhibit robust signs of Parkinsonism despite showing selective disruption of AMPK-PGC-1α axis in their ventral midbrain. It is likely that a combination of extrinsic and intrinsic factors is involved in mediating overt neuronal death, as we and others have previously documented (Zhang et al. 2017; Sulzer 2007), and that AMPK deficiency represents one of these contributing factors to PD pathogenesis.

It is noteworthy to highlight that several groups have found that AMPK activation could confer neuroprotection in PD models. For example, Choi and colleagues demonstrated that AMPK is activated in mice treated with MPTP and that inhibition of AMPK function by compound C enhances MPP(+)-induced cell death (Choi et al. 2010). Similar findings were made in another recent study albeit in cultured cells exposed to rotenone (Wu et al. 2011). However, in contradiction to these findings, Kim et al. found that AMPK mediates the atrophy of DA neurons in mice exposed to 6-hydroxydopamine (6-OHDA) (Kim et al. 2013). In a related study, Xu et al. observed similar detrimental effects of AMPK activation in primary neurons treated with 6-OHDA, MPP+, or rotenone (Xu et al. 2014). Thus, the role of AMPK in neuroprotection remains controversial. Notwithstanding this, our own study conducted previously in *Drosophila* genetic models of PD demonstrating that AMPK activation ameliorates the pathological phenotypes of the mutant flies would favor a neuroprotective function of AMPK. Furthermore, a cohort-based study involving 800,000 individuals revealed that Metformin-inclusive sulfonylurea therapy significantly reduces the risk for the disease occurring with Type 2 diabetes in a Taiwanese population (Wahlqvist et al. 2012), suggesting that AMPK activation is beneficial for PD. Related to this, a recent system of biology-based study identified PGC-1α as a potential therapeutic focus for intervention in PD (Zheng et al. 2010). Notably, the authors found that bioenergetic genes responsive to PGC-1α are under-expressed in patients with PD, suggesting that the upregulation of PGC-1α may be beneficial.

Given that AMPK is a potent upstream activator of PGC-1α, it is intuitive to propose that pharmacological enhancement of AMPK-PGC-1α activity would represent a rational therapeutic strategy. Interestingly, Martin-Montalvo et al. recently demonstrated that prolonged treatment of mice with metformin significantly improves both their lifespan and health span, although the brain AMPK profile was not examined (Martin-Montalvo et al. 2013). Using similar treatment paradigm, we showed that metformin administration promotes the activation of AMPK (and concomitantly PGC-1α expression) selectively in ventral midbrain region of Parkin null mice that effectively restore the otherwise deficient pAMPK level in these mutant mice. Interestingly, we observed that the upregulation of AMPK-PGC-1α pathway took place against the backdrop of an enhanced PARIS expression in the metformin-treated group. Although PARIS functions as a negative regulator of PGC-1α expression, pharmacological AMPK activation alone is clearly sufficient to circumvent the repression by PARIS to result in the restoration of the deficient AMPK-PGC-1α axis in Parkin null mice. Overall, our finding extended the study by Martin-Montalvo et al. (2013) and provides support to the above-mentioned Taiwanese cohort study that documented a reduced risk for metformin-treated diabetic patients to develop PD (Wahlqvist et al. 2012).

In conclusion, the main finding of our current study is that the AMPK activation is physiologically upregulated in the mammalian ventral midbrain and selectively reduced in the same region in the absence of Parkin or PINK1, the deficiency of which may contribute to SNpc DA neurodegeneration in Parkin/PINK1-related PD cases. Alongside this, we also found that the deficiency in midbrain AMPK expression may be corrected by pharmacological treatment with AMPK activators.
